# A Promising Listeria-Vectored Vaccine Induces Th1-Type Immune Responses and Confers Protection Against Tuberculosis

**DOI:** 10.3389/fcimb.2017.00407

**Published:** 2017-09-28

**Authors:** Yuelan Yin, Kai Lian, Dan Zhao, Chengwu Tao, Xiang Chen, Weijun Tan, Xiaobo Wang, Zhengzhong Xu, Maozhi Hu, Yan Rao, Xiaohui Zhou, Zhiming Pan, Xiaoming Zhang, Xin'an Jiao

**Affiliations:** ^1^Jiangsu Key Laboratory of Zoonosis, Joint International Research Laboratory of Agriculture and Agri-Product Safety, Jiangsu Co-Innovation Center for Prevention and Control of Important Animal Infectious Disease and Zoonosis, Yangzhou University, Yangzhou, Yangzhou, China; ^2^ABSL-3 Lab, Wuhan University, Wuhan, China; ^3^Department of Pathobiology and Veterinary Science, University of Connecticut, Storrs, CT, United States; ^4^Institut Pasteur of Shanghai, Chinese Academy of Sciences, Shanghai, China

**Keywords:** *Mycobacterium tuberculosis*, Th1/Th17, protective efficacy, *Listeria monocytogenes*, attenuated, FbpB-ESAT-6

## Abstract

Deaths associated with tuberculosis (TB) is rising and accounted for 1.4 million deaths in 2015 many of which were due to drug-resistant bacteria. Vaccines represent an important medical intervention, but the current Bacilli Calmette-Guerin (BCG) vaccine is not ideal for the protection of teenagers and adults. Therefore, a safe and effective vaccine is urgently needed. In this study, we designed a novel vaccine using an attenuated *Listeria monocytogenes* strain carrying fusion antigen FbpB-ESAT-6 (rLM) and characterized its safety and protective efficacy against *Mycobacterium tuberculosis* (*M.tb*) infection in mice. Compared to the wild type strain yzuLM4 and parental strain LMΔ*actA/plcB* (LM1-2), the virulence of rLM was significantly reduced as judged by its infectious kinetics and LD_50_ dose. Further characterization of intravenous immunization showed that prime-boost vaccination significantly increased the levels of Th1 cytokines (IFN-γ, IL-17, and IL-6), and enhanced cytotoxic T lymphocyte (CTL) CTLs activity, suggesting that rLM could elicit potent Th1/Th17 responses. More importantly, rLM significantly conferred the protection against *M.tb* H37Rv challenge. Collectively, our findings indicated that rLM is a novel and useful tool to prevent *M.tb* infection, and can be potentially be used to boost BCG-primed immunity.

## Introduction

Tuberculosis (TB) is an infectious disease caused by the *Mycobacterium tuberculosis* (*M.tb*) complex. TB incidence has fallen by an average of 1.5% per year since 2000, yet during the past few years, the proportion of multidrug-resistant TB (MDR-TB) and extensively drug-resistant TB (XDR-TB) cases has increased steadily (World Health Organization, 2016). Therefore, it is highly urgent to control TB occurrence. However, effective prevention of the infection, recurrence and reactivation of latent TB remain to be significant challenge. Vaccination is an important medical intervention in TB prevention strategies. Bacilli Calmette-Guerin (BCG) is the only vaccine approved worldwide for clinical use to prevent TB, but it fails to induce enough *M.tb*-specific CD8^+^ T cells and elicit optimal immune memory in the lung (Beverley et al., 2014). Its efficacy is variable, ranging from 0 to 80% in different regions, and it is not ideal for the protection of teenagers or adults (Henao-Tamayo et al., 2014). TB vaccine developers are seeking new ways to rationally and efficiently select candidates for human efficacy trials.

Host immune response against *M.tb* is mainly mediated by cellular immunity, in which Th1 cytokines IFN-γ, TNF-α are absolutely required to control bacterial growth (Cavalcanti et al., 2012; Kim et al., 2014). IL-17 secreted by Th17 cells can accelerate the initial response and promote the recruitment of Th1 cells to the site of infection, contributes to vaccine efficacy (Torrado and Cooper, 2010). IL-6 contributes to host resistance by its proinflammatory activity and by its influence on cytokine secretion. While anti-inflammatory cytokine IL-4 and IL-10 secreted by Th2 CD4 cells down-regulate the Th1 response, lead to pulmonary fibrosis (Rook et al., 2004). Effective TB vaccines will need to enhance Th1/Th17 immunity and suppress pre-existing Th2-like activity. Attenuated *Listeria monocytogenes* (LM), as an immune modulator and vaccine adjuvant, induces a strong cellular immunity characterized by Th1-type CD4 T cell and CD8 T cell activation, has been successfully developed and used as a vaccine carrier and is a attractive TB vaccine vehicle for delivering *M.tb* antigens.

Because LM has a unique intracellular parasitic life (Decatur and Portnoy, 2000), it can be used as a promising adjuvant to induce the generation of innate immunity, through the secretion of various critically important cytokines, e.g., IFN-γ, IL-12, and IL-18 (Nomura et al., 2002), and the generation of adaptive immunity by preferentially promoting the proliferation of antigen-specific CD4^+^ T cells and CD8^+^ T cells. The secreted protein listeriolysin O (LLO) encoded by *hly* is prominent in generating the Th1 immune response (Yamamoto et al., 2005; Kono et al., 2012). Notably, Grode et al. successfully expressed LLO in BCGΔureC::*hly* (VPM1002), leading to superior protective efficacy (Grode et al., 2005). Gunn et al. fused LLO with HPV 16 E7 protein (Lm-LLO-E7), which can augment the E7-specific CD8^+^ T cell response and repressing murine tumors (Gunn et al., 2001). We have reported that the LLO-esat-6 fusion protein delivered by attenuated LM significantly elicited a strong antigen-specific T cell-mediated immune response (Yin et al., 2012).

The safety of LM vaccine has been well-demonstrated in human clinical trials: Angelakopoulos's clinical trial demonstrated the ability of an attenuated LM-based empty vaccine to safely and effectively induce antigen-specific T cell responses in humans (Angelakopoulos et al., 2002); Maciag's Phase I trial and Petit's Phase II clinical trial with LM-LLO-E7 verified that the safe and effective recombinant vaccines could be potentially a new therapeutic option (Maciag et al., 2009; Petit and Basu, 2013). LM has particular advantages to offer as a neonatal vaccine vehicle: attenuated strains of LM that are safe for neonates have now been identified, and interestingly, they are very efficient at inducing robust Th1-type immunity in neonates (Kollmann et al., 2007).

LM has been successfully developed as a novel live vaccine vector, especially for tumor immunotherapy. However, LM-based anti-*M.tb* vaccines are rarely reported. Both our previous report (Yin et al., 2012) and other studies (Miki et al., 2004; Lin et al., 2015) suggested that a *Listeria*-based vaccine could potentially prevent TB. In this study, protective antigens ESAT-6 and FbpB with multiple T cell epitopes and B cell epitopes were chosen as delivering targets (D'Souza et al., 2003; Brodin et al., 2006), the fusion protein FbpB-ESAT-6 was expressed and characterized the innate and adaptive immune response induced by *Listeria*-based TB vaccine and determined its protective efficacy against wild type *M.tb* challenge.

## Materials and methods

### Bacteria

Virulent serotype 1/2a LM strain yzuLM4 and attenuated LM strain yzuLM4Δ*actA/plcB* (LM1-2) were cultured in brain heart infusion (BHI) broth (Yin et al., 2008). *M.tb* H37Rv was preserved in an ABSL-3 lab at Wuhan University. BCG vaccine was purchased from the Yangzhou Center for Disease Control and Prevention.

### Experimental animals

Six-week-old female C57BL/6 mice (H-2^b^) were purchased from Vital River Laboratory Animal Technology Co., Ltd. (Beijing, China). All animals were immunized at the animal biosafety facilities and all procedures were approved by the institutional animal ethics approval committee of Yangzhou University. Animals received free access to water and commercial mouse chow throughout the study. The challenge experiments were conducted in an ABSL-3 lab at Wuhan University.

### Construction of the recombinant strain

To achieve homologous recombination, the recombinant plasmid pKSV7-*act*A*/fbpB-esat-6*/*plc*B was constructed (Figure S1) with primers pairs showing in Table S1, in which the *fbpB* gene (850 bp) was flanked with a DNA fragment consisting of the 698-bp upstream fragment of *actA* and signal peptide sequence of *actA* (75 bp). The *esat-6* gene was flanked by the downstream sequence of the *plcB* gene (861bp). A 30-bp linker encoding (Gly4Ser) was designed between *fbpB* and *esat-6* (285 bp). The recombinant plasmid pKSV7-*act*A/*fbpB-esat-6*/*plc*B was electroporated into competent LM1-2 cells. The recombinant strain LM::*fbpB-esat-6* (rLM) was obtained according to previously described methods (Watanabe-Takano et al., 2014). The positive clones were verified by sequencing.

### Western blotting

Secreted proteins were precipitated from culture supernatants using trichloroacetic acid (TCA) and run on a sodium dodecyl sulfate polyacrylamide gel. After transfer to a polyvinylidene difluoride (PVDF) membrane (NEN Life Sciences, Boston, MA) using a Western blot apparatus (Bio-Rad, Melville, NY), the proteins were incubated with a rabbit anti-ESAT-6 monoclonal antibody (HYB-076-08, Abcam, Hong Kong, China) and then stained with a peroxidase-labeled anti-rabbit antibody (BD Pharmingen, USA).

### Virulence of listeria strains *in vivo*

The LD_50_ of listerial strains was determined with groups of mice intravenously inoculated with 0.1 mL of an appropriate three-fold dilution of the recombinant or parent strain in PBS. Surplus bacteria were plated on BHI agar plates for bacterial counts. The mice were monitored for the next 14 days, and the median lethal dose (LD_50_) was determined. Bacterial enumeration in spleens and livers of mice was evaluated via intravenously injected with 0.1 LD_50_ of rLM or LM1-2 on day 1, 3, 5, and 7.

### Cytokine secretion assay

Six-week-old C57BL/6 mice were randomly divided into five groups (five mice/group). Three groups were prime-boost immunized i.v. with of rLM (0.1LD50), LM1-2 (0.1LD50), and 100 μl PBS, respectively; the fourth group was immunized with BCG (1 × 10^6^ CFU) i.v.; the fifth group was primely immunized with BCG (1 × 10^6^ CFU), and boosted with rLM (0.1LD50). A total of 1 × 10^6^ murine splenic cells lymphocytes from C57BL/6 mice were added onto 96-well microplates, The stimulator ESAT-6_1–20_ and FbpB_240–259_ (final concentration 5 μg/ml), purified protein derivative (PPD, Switzerland Prionics company, final concentration10 μg/ml), and ConA (final concentration 10 μg/ml) was co-incubated with these lymphocytes, respectively. Supernatants from each group were harvested and detected by mouse inflammation kit and mouse Th1/Th2/Th17 cytokine kit (R&D, USA) according to manufacturers' instructions.

### Cytotoxicity assay using the CFSE fluorescent-based dye

To determine the cytotoxicity *in vivo*, the same immunization procedure was used as described as cytokine secretion assay. On day 8 after boost immunization, spleen cells were pooled from naive C57BL/6 mice and divided into two groups; the cell suspension for one group was incubated with PPD at 37°C for 45 min and subsequently labeled with CFSE^high^ (2.5 μM, Invitrogen, USA) buffer at 37°C for 10 min, the other group was incubated without PPD and labeled with CFSE^low^ (0.25 μM) buffer, 1 × 10^7^ mixed cells were intravenously injected into mice that had previously prime-boost immunized with rLM, LM1-2, BCG, and PBS, respectively, and 15 h later, cell suspensions from spleen of mice were analyzed by flow cytometry, and each population was analyzed by a FACScan in which each population was detected according to the differing CFSE fluorescence intensities. To calculate specific lysis, the following formula was used: The percentage of specific lysis was calculated using the following formula: percent specific lysis = 100 − [100x (% CFSE_high_ immunized/% CFSE_low_ immunized)/(% CFSE_high_ control/% CFSE_low_ control)].

### *M.tb* challenge

For challenge experiments, 6-week-old female C57BL/6 mice were randomly divided into five groups (eight mice/group), four groups were prime-boost immunized at 14 days interval, including PBS group (100 μl), LM1-2 group (1 × 10^6^ CFU), rLM (2 × 10^7^ CFU), BCG/rLM group (primary vaccinated with BCG, boosted with rLM). The fifth group was BCG single immunized group (1 × 10^6^ CFU). Twenty-eight days after second immunization, mice were challenged with 5 × 10^5^ CFU virulent *M.tb* H37Rv via a lateral tail vein, the inoculum plated at the time of challenge. Six weeks post-challenge, all mice were sacrificed. The spleens and livers of five mice per group were removed, homogenized and cultured for CFU of *M.tb*. The left lung of each mouse was excised and fixed in 10% phosphate-buffered formalin, sectioned, stained with hematoxylin and eosin, and examined for histological lesions with microscope. Alternatively, the tissues were subjected to acid-fast staining to visualize the bacilli. The challenge experiment was conducted twice.

### Statistical analysis

Differences between groups were analyzed using the Statistical Package for Social Sciences software (SPSS version 15.0; SPSS, Chicago, IL, USA). *P* < 0.05 were considered significant, and values <0.01 were considered highly significant.

## Results

### Expressed fusion protein FbpB-ESAT-6 retains hemolytic activity and immunogenicity

**Figure 1:**
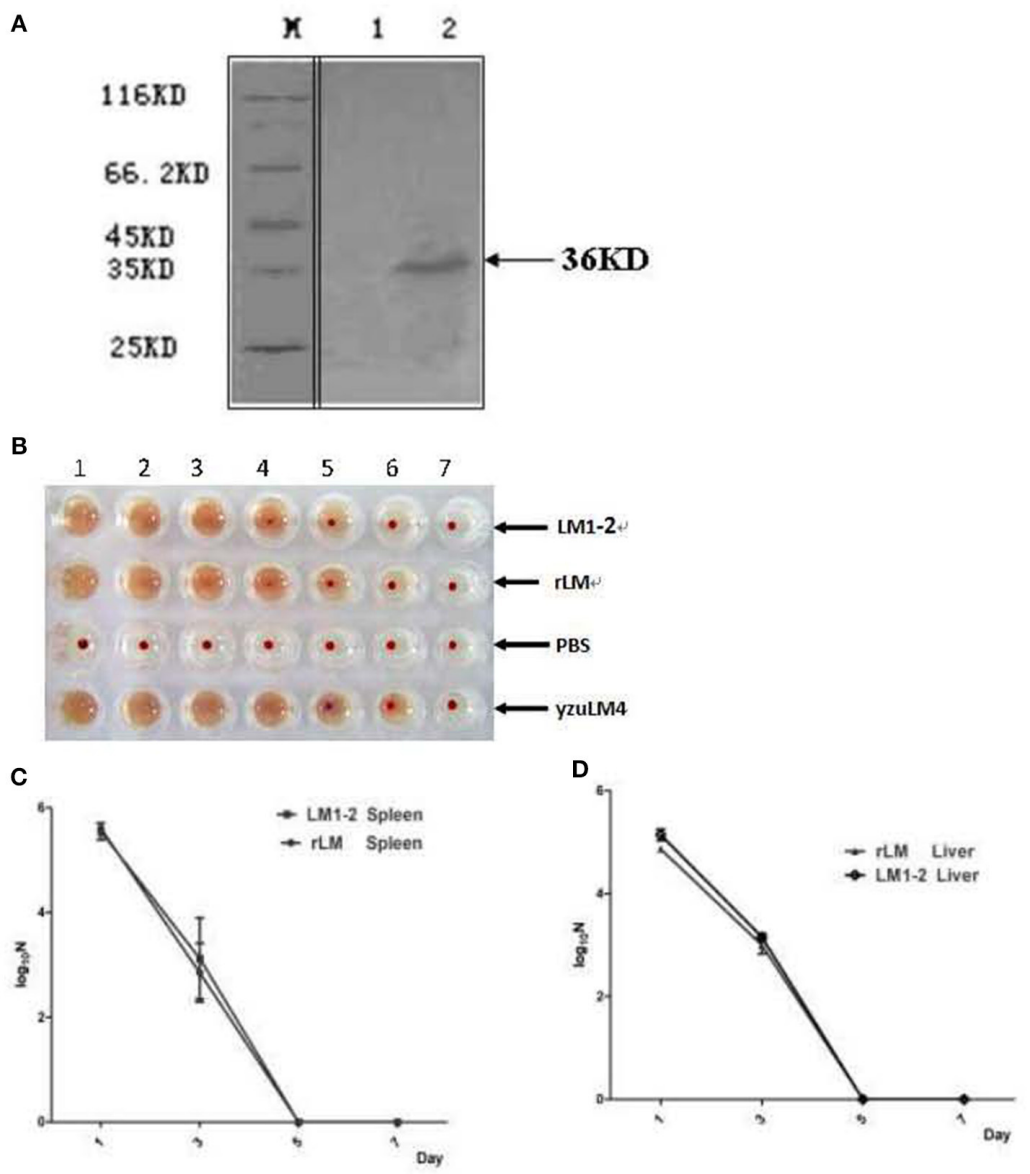
Identification of recombinant *L. monocytogenes* strain. **(A)** Western blot analysis of expressed FbpB-ESAT-6 protein. Secreted proteins were TCA-precipitated and probed by a rabbit anti-ESAT-6 monoclonal antibody by western blot. M represents protein marker; lane 1 represents proteins from LM1-2, lane 2 represents the proteins from rLM. **(B)** Hemolytic activity of listeria strains. Culture supernatants were incubated with sheep erythrocytes at 37°C in two-fold serial dilutions (wells 1–7). The recombinant strain rLM had hemolysis titers of 2^3^, while the parental strains were 2^4^. **(C,D)** Kinetics of different LM strains during infection in spleens **(C)** and livers **(D)** of mice. C57BL/6 mice (20 mice per group) were injected intravenously (i.v.) with rLM and LM1-2 at a dose of 0.1 LD_50_, respectively. Bacteria in spleen and liver were enumerated on day 1, 3, 5, and 7 post-inoculation by tissue homogenization and serial plating.

A recombinant *L. monocytogenes* strain LM::*fbpB-esat-6* was successfully constructed via homologous recombination technology, the secretive expression of FbpB-ESAT-6 was verified by Western blotting using rabbit anti-ESAT-6 monoclonal antibody. The appearance of the specific 36 kDa target band indicated that fusion protein Ag85B-ESAT-6 secretively expressed and had an effective immunoreactivity (Figure 1A). LLO provides LM unique adjuvant character of generating Th1 immune response, the hemolysis activity of rLM was performed, as shown in (Figure 1B), the hemolysis titer of the recombinant strains reached 2^3^, which was lower than that of the parental strains yuzLM4 (2^4^), thus rLM displayed good hemolytic activity.

### The virulence of recombinant LM strain was reduced in mice

Novel vaccine candidates are aimed at prevented human tuberculosis, the safety of rLM is firstly considered and evaluated by bacterial growth kinetics and LD_50_ determination. The bacterial growth kinetics *in vivo* showed that there was no significant difference in livers and spleens of mice in rLM infection group comparing with LM1-2 group (*P* > 0.05) on each detection point, indicated that the translocation ability of the recombinant strain was reduced and the virulence is decreased (Figures 1C,D). The notable reduction of virulence was shown in Table 1, the recombinant strain was reduced by 4.67 × 10^4^- and 4.9-fold comparing with wild type strain yzuLM4 and its parental strain LM1-2, respectively. These results suggested that the safety of the recombinant strain was distinctly improved.

**Table 1:** LD_50_ of recombinant, parental, and wild type strains.

**Dose (CFU)**	**yzuLM4 (1** × **10**^**4**^**)**				**LM1-2 (1** × **10**^**7**^**)**				**rLM (1** × **10**^**8**^**)**			
CFU per mouse	15.03	5.01	1.67	0.56	10.53	3.51	1.17	0.39	29.3	9.75	3.25	1.08
Mortality	5/5	5/5	5/5	2/5	5/5	1/5	0/5	0/5	5/5	5/5	3/5	0/5
LD_50_	6.23 × 10^3^ CFU				4.17 × 10^7^ CFU				2.90 × 10^8^ CFU			

*C57BL/6 mice (five mice per group) were injected i.v. with increasing doses of recombinant and parental strains. The mice were monitored for the next 14 days and data were calculated by the Reed-Muench method*.

### Pro- and anti-inflammatory cytokine responses among groups

**Figure 2:**
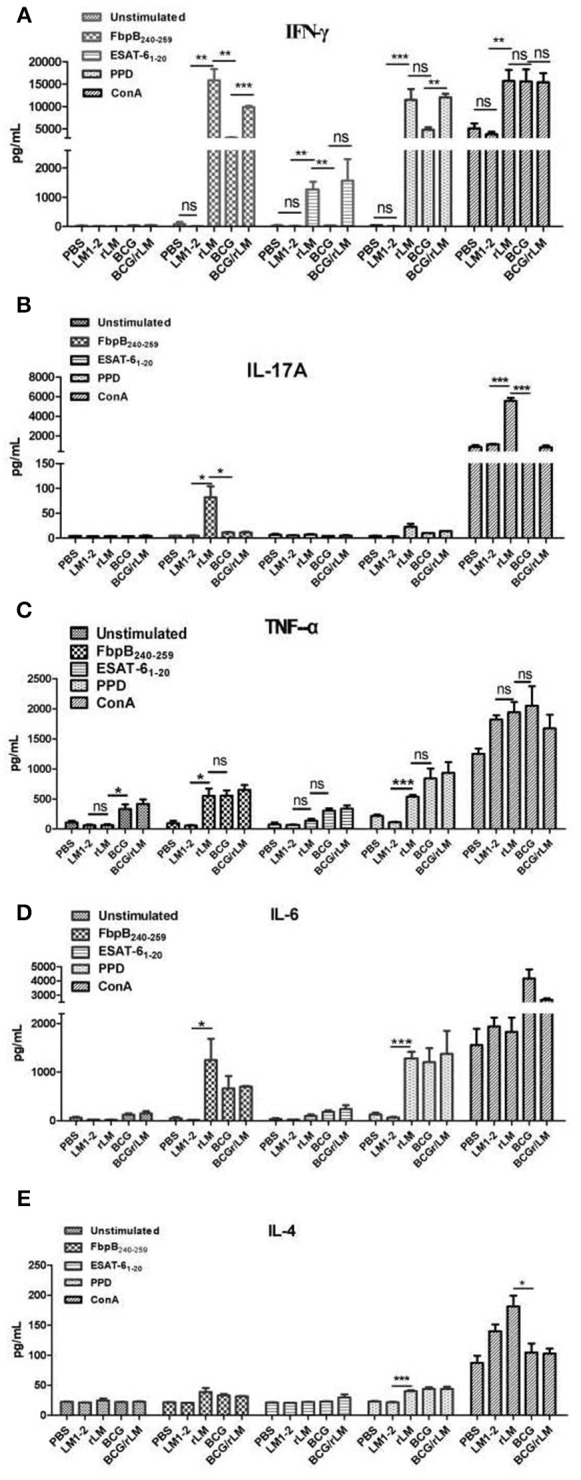
The level of inflammatory cytokines in the splenocytes of mice immunized with indicated LM strains or controls. Five groups of C57BL/6 mice (*n* = 5) were intravenously immunized with rLM, LM1-2, PBS, BCG, and BCG/rLM, respectively. IFN-γ **(A)**, IL-17A **(B)**, TNF-α **(C)**, IL-6 **(D)**, and IL-4 **(E)** were determined on the 8th day after immunization. Splenocytes were prepared and stimulated with peptide FbpB_240−259_, ESAT-6_1−20_, PPD and ConA. Cytokines in the supernatants from each well were determined by ELISA. The results were presented as the mean ± SD of three wells in triplicate. ^***^indicated *P* < 0.001, ^**^indicated *P* < 0.01, and ^*^indicated *P* < 0.05 of the comparisons. **(A–E)** Cytokines in the splenocytes.

Pro-inflammatory cytokine IFN-γ is crucial for the immune response to *M.tb*, and IL-17 contributes antibacterial infection. The results illustrated in Figure 2 showed that significantly higher levels of IFN-γ and IL-17A were induced by vaccination with rLM than the negative control group (*P* < 0.01; *P* < 0.05) and BCG group (*P* < 0.01; *P* < 0.05) in spleens (Figures 2A,B), significantly higher levels of TNF-α and IL-6 (Figures 2C,D) than the negative control group (*P* < 0.05); while low level of IL-4 were induced in each group (Figure 2E), and there was no significant difference among groups except PPD as stimulator. These results indicated that Th1/Th17 cellular immunity was elicited by rLM.

### rLM strain elicited strong CTL immune responses

**Figure 3:**
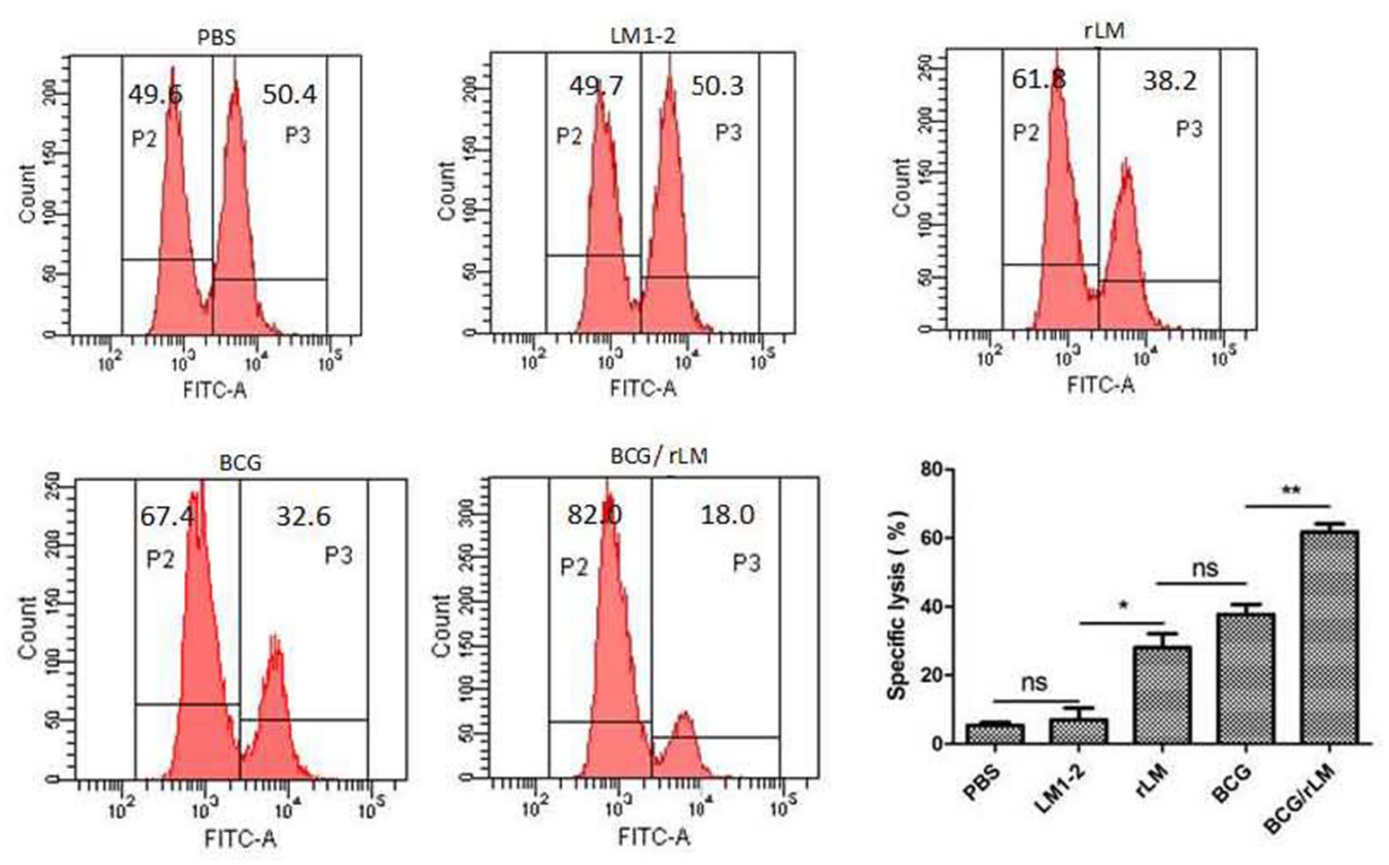
PPD-specific CTL responses *in vivo*. PPD-specific CTL activity *in vivo* in mice (*n* = 5 per group) immunized with rLM, LM1-2, PBS, BCG, or BCG/rLM, respectively. A mixture of CFSE-labeled splenocytes pulsed with PPD (CFSE^high^) or without PPD(CFSE^low^) was intravenously injected into each group of mice on day 7 after the second immunization. The histogram shows CFSE^low^ untreated and CFSE^high^ PPD pulsed target cells in the spleens of mice after 15 h of transfer, respectively. Results were representative of three experiments, each with five mice per group. ^*^P < 0.05 rLM group vs. LM1-2 group; ^**^P < 0.01 BCG/rLM group vs. BCG group.

CTL plays a very important role in cellular immune responses, conducing to eradicate of intracellular paracitical *M. tuberculosis*. Mice immunized with rLM were able to produce efficiently PPD-specific cytotoxic T lymphocytes than the negative control group (*P* < 0.05) (Figure 3). Additionally, the killing effect of the group with combined immunization of both BCG and rLM (BCG/rLM) was stronger than BCG immunized group (*P* < 0.01). These results indicated that rLM strain could elicite strong CTL immune responses against *M.tb* antigens. The strong CTL immune responses elicited by rLM were predicted to enhance the clearance of *M.tb*.

### Protective efficacy of prime-boost rLM

#### Enhanced protection by BCG prime-rLM boost

**Figure 4:**
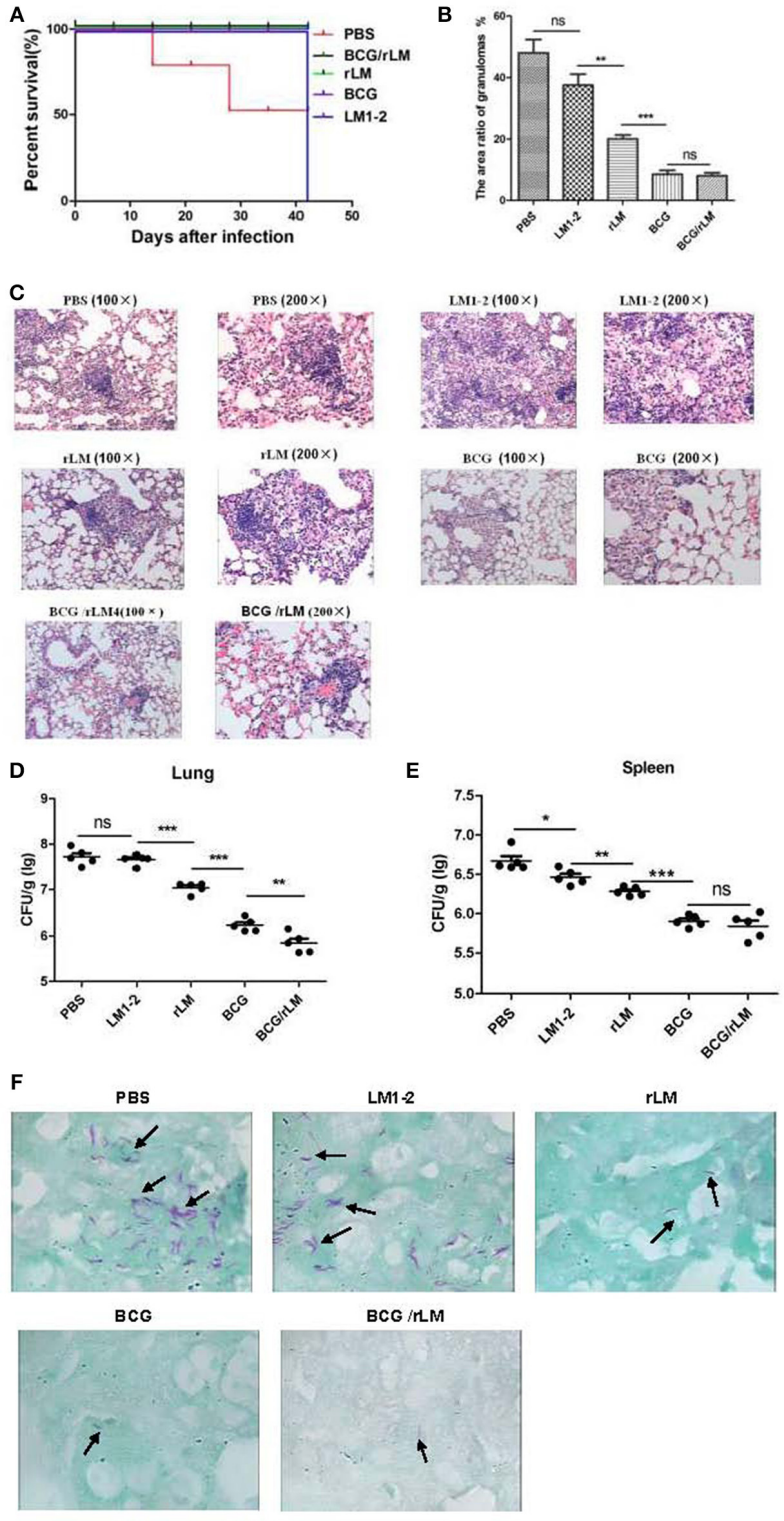
Protective efficacy of prime-boost vaccination with rLM. After 42 days of immunization, mice were challenged with 5 × 10^5^ CFU virulent *M.tb* H37Rv via a lateral tail vein. Six weeks post-challenge, all mice were sacrificed. The spleens and livers of five mice per group were removed, homogenized and cultured for CFU of *M.tb*. The left lung of each mouse was excised and fixed in 10% phosphate-buffered formalin, sectioned, stained with hematoxylin and eosin, and examined for histological lesions with microscope. Alternatively, the tissues were subjected to acid-fast staining to visualize the bacilli. The challenge experiment was conducted twice. **(A)** Survival of the immunized mice challenged by *M.tb* H37Rv. **(B)** The area ratio of granulomas per slice in each group of mice; **(C)** Histopathology (HandE staining) of the spleen in mice in each group; **(D,E)** Bacteria load in lung **(D)** and spleen **(E)** of mice. **(F)** Lung tissue with anti-acid staining.

The vaccination and challenge procedure was shown in Figure 4A. The result of immune protective efficacy showed that two mice died in the PBS group on day 14 and 28, respectively, and one mouse died on day 28 in the LM1-2 vaccination group. In the other three groups, all the mice survived, and the protective efficacy of these three groups was 100% (Figure 4A).

#### Reduced pathology following prime-boost with rLM

The bacterial load and histopathological assays suggested that the prime-boost rLM not only reduced the bacterial load, but also conferred superior protection against pathological damage and granuloma formation (Figures 4B,C). It was interesting that the LM1-2-vaccinated group reduced granuloma inflammation in the lung by 10.5% (Figure 4B). The combination vaccine BCG prime-rLM boost strategy showed superior protection against TB.

#### Decreased bacterial load in the lung

The prime-boosted rLM group significantly reduced the bacterial load in the lung compared with LM1-2 group (*p* < 0.01). The bacterial load in the lungs of mice vaccinated with BCG/rLM was significantly lower than in the BCG group (*p* < 0.01) (Figures 4D,E). The Bacilli observed in tissues subjected to acid-fast staining was consistent with bacterial load calculation in the lung and spleen (Figure 4F). Thus, these results suggested that rLM could confer protection against *M.tb* challenge.

## Discussion

TB is a serious worldwide threat to public health security, and it is urgent to design and develop novel TB vaccines. Novel approaches to TB vaccination are developing rapidly, and 17 vaccine candidates are in the clinical trial (Garg et al., 2014). However, how to enhance the synergistic action between CD4^+^- and CD8^+^-T cells remains worth exploring (Principi and Esposito, 2015; Shang et al., 2016). LM as an intracellular pathogenic antigen carrier, has the advantage of inducing multivalent innate immunity as well as a cell-mediated immune response (Jiang et al., 2007). The advantage of LM inducing strong protective immune responses has been intensively studied in molecular biology and immunology (Condotta et al., 2012). It is an effective and promising vaccine vehicle for tumor immunotherapy. Based on the safety and immunity investigation of our previous study on LM::*esat-6*, the vector potentiality of the highly attenuated LM for delivering the *M.tb* antigens FbpB and ESAT-6 was further probed.

It is worth mentioning that CD8^+^ T cells are believed to play a central role in the control and obliteration of intracellular pathogens (Agnellini et al., 2008; Condotta et al., 2012). The major functional subset of CD8^+^ T cells is CD8^+^ cytotoxic lymphocytes (CTL), which mediates the cellular immune response to infected cells, contributing to the clearance of intracellular pathogens and infected cells (Gallichan and Rosenthal, 1996; Brighenti and Andersson, 2012). In this study, we determined the potential ability of rLM induced CD8^+^ cytotoxic lymphocytes activity. Tuberculin purified protein derivative (PPD) was used as stimulator for cytotoxicity assay, it has been verified by LC-MS/MS that Ag85B and ESAT-6 are the components of PPD (Borsuk et al., 2009). The result showed that elevated mycobacterial antigen-specific CTL immune responses were elicited by rLM delivering fusion antigen Ag85B-ESAT-6. It is well recognized that CTL activation correlates with the Th1 immune response (Huang et al., 2007). Additionally, significantly higher levels of pro-inflammatory cytokines IFN-γ, TNF-α, IL-17, and IL-6 were elicited by vaccinating with rLM, while lower levels of anti-inflammatory cytokine IL-4 was produced; on one hand, these pro-inflammatory factors play important roles in assisting and augmenting CD8^+^ T cell immunity (Pamer, 2004); on the other hand, they improve the development of Th1 cells and contribute to the host response to mycobacteria. Among these pro-inflammatory cytokines, IFN-γ, and TNF-α are considered to perform the major role of Th1 immune responses in protective immunity against TB (McNab et al., 2015). IL-17 attracts the recruitment of Th1 cells to the site of infection, contributes antibacterial infection (Xu et al., 2010). IL-6 producing by T cells and macrophages drives Th17 cells polarization, stimulates immune response, plays a role in fighting infection (Ladel et al., 1997). We consider that strong Th1 immune response, the strong CD8^+^ cytotoxic lymphocyte killing activity, heightened pro-inflammatory cytokines production and decreased anti-infammatory cytokines to have elevated the protection against high-dose *M.tb* H37Rv challenge via the intravenous route.

Th17 cells, differentiated from naïve CD4^+^ T cells, are the major cell type producing IL-17A (Xu et al., 2010). The importance of IL-17A in inherent immunity is well known, while its importance in adaptive immunity and protective immunity for *M.tb* infection has not received enough attention until recent years (Khader and Gopal, 2010). Verwaerde's report found that a lack of significant IL-17 production in the spleen and lung resulted in no significant protection after HBHA vaccination (Verwaerde et al., 2014). T cell immunity after vaccination with M72/AS01 in South African adults showed that most vaccination-induced T cells did not express Th1 cytokine or IL-17 (Day et al., 2013). Gopal's study verified that early protective immunity against hypervirulent *M.tb* HN878 requires IL-17 (Gopal et al., 2014). Therefore, the Th17 immune response is an important goal for an effective vaccine against TB (Muranski et al., 2011). In this study, the IL-17A production levels of Th17 cells in the spleen of mice were determined, and the results indicated that BCG vaccination can't induce obviously IL-17 secretion in splenic lymphocyte stimulated with PPD or FbpB_240-259_, this result was consistent with Garcia-Pelayo's report (Garcia-Pelayo et al., 2015), while rLM could induce significantly FbpB-ESAT-6-specific IL-17^+^ T cell immune responses, it is consistent with Xu's report of a *Listeria* vector delivering OVA which verified that Th17 cells promote the CTL response and that IL-17A is required for the generation of an Ag-specific CD8^+^ T cell response against primary infection (Xu et al., 2010). Based on the above-mentioned reports and our results, we deduce that Th1/Th17 immune responses elicited by vaccination with rLM were crucial for the reduction in the bacterial load and in the inflammatory lung lesions caused by *M.tb* challenge in C57BL/6/c mice and could afford 100% protection efficacy against *M.tb* H37Rv.

BCG strains lack numerous *M.tb* antigens, some epitopes sequence variants have not been maintained in the BCG strains, including epitopes derived from *fbpB, mce*, and *hemK* (Copin et al., 2014). Moreover, T cell antigens such as ESAT-6, CFP10, and PPE68 are absent from BCG strains, which are the main reason for BCG's failure to induce sufficient cellular immune protection (Copin et al., 2014). Secretory proteins ESAT-6 and FbpB (Ag85B) with multiple T cell epitopes and B cell epitopes are crucial protective antigens (D'Souza et al., 2003; Brodin et al., 2006). In this study, the fusion protein FbpB-ESAT-6 was expressed and secreted by a *Listeria* vector and could induce strong Th1-type immune responses and confer efficient protection against high doses of *M.tb* H37Rv intravenous infection. rLM could induce of Th1-type immune response, thus it is a novel attractive vaccine candidate with clear advantages. Finally, with minor details needing to be finalized, the peptide ESAT-6_1−20_ stimulation did not generate the most robust immune responses comparing with FbpB_240−259_, it is possibly related with the construction strategy, ESAT-6 was fused to FbpB with linker of Gly-Gly-Gly-Gly-Ser, the amino acid sequence is downstream of FbpB, 3D structure may be partly affected to the natural epitopes of ESAT-6. The optimization of order, linker, and copies of two antigens deserves further exploration to improve the induction of antigen specific cellular immunity.

Because novel vaccine candidates are intended to prevent human tuberculosis in clinical use, safety is a first consideration. Accordingly, we chose a highly attenuated serovar, 1/2a *L. monocytogenes* with deletion of *actA* (75-bp signal sequence maintained) and *plcB* as the parental strain. Its 50% lethal dose in mice was increased 46,700-fold compared to the parent strain. Moreover, the serotype of strain LM1-2 is the same as for attenuated *Lm* H1169 with deletion of 49% of *actA* and 82% of *plcB*. A study of the safety of H1169 by Angelakopoulos et al demonstrated that *actA*/*plcB*-deleted attenuated LM may be administered without serious sequelae to carefully monitored adult volunteers (Angelakopoulos et al., 2002). The enhanced safety of this recombinant TB vaccine candidate lays a strong foundation for the evaluation of clinical efficacy. More importantly, our reports verified that the deletion of both *actA* and *plcB* allows the induction of robust T cell responses in mouse models.

In summary, our data demonstrated that rLM remarkably increased the levels of Th1 cytokine and the activity of CTLs. Moreover, rLM could enhance the vaccination effects and significantly elevate protection against *M.tb* H37Rv challenge, e.g., reducing the bacterial burden and pathological lesions in the lungs and spleen. More importantly, the safety profile of the recombinant strain is encouraging. Collectively, our findings indicated that rLM could compensate the limitations of BCG, as a novel and useful tool for preventing *M.tb* infection, and a promising vaccine used to boost BCG-primed immunity.

## Author contributions

YY and KL made main part of all experiments, and YY finished written the manuscript. DZ, CT, and WT took part in the immune analysis experiments. XC and XW participated in pathological observation. ZX and MH had a hand in analysis with flow cytometry. XiaohZ, ZP, and XiaomZ guided the implement of the experiments, XiaohZ also revised the manuscript, XiaomZ guided the operation related with labeled and analyzed with FACS. YR took part in challenge experiments. XJ guided the implement of all experiments, and supported the funds.

### Conflict of interest statement

The authors declare that the research was conducted in the absence of any commercial or financial relationships that could be construed as a potential conflict of interest.
