# Supplementary material for: A Promising Listeria-Vectored Vaccine Induces Th1-Type Immune Responses and Confers Protection Against Tuberculosis
Source: Front Cell Infect Microbiol. 2017 Sep 28;7:407. doi: 10.3389/fcimb.2017.00407 (PMC5626977; doi:10.3389/fcimb.2017.00407)
Supplement: Supplementary file 1 [file DataSheet1.docx]

Supplement Figure 1 Schematic diagram of constructing the recombinant strain rLM.

LM1-2 was constructed with deletion of 1845-bp of *actA* and 790-bp of *plcB* in yzuLM4. rLMlm was constructed with insertion of fusion genes *fbpB*-*esat-6* between truncated *actA* and *plcB* in the genome of LM1-2.

Supplement Table 1 Primers pairs used to amplify related genes

| primers | sequence | length |
| --- | --- | --- |
| *act*A1 | 5’ -GGGAATTCCGAGTCGTTCGCCGATGTTT-3 | 698bp |
| *act*A2 | 5’-AGCCCGGCCGGGAGAATGCAAATATTATGTCGGGGTTAA-3 |  |
| *fbp*B1 | 5’-TAACCCCGACATAATATTTGCATTCTCCCGGCCGGGGCT-3 | 850bp |
| *fbp*B2 | 5’ -AAGGATCCACCGCCACCGCCGGCGCCTAACGAA-3 |  |
| *esat-61* | 5’-AAGGATCCGTTGGCGGTGGCTCCATGACAGAGCAGCA-3 | 285bp |
| *esat-62* | 5’ -TTAGTCTAGCTCCAGTAGGCTATGCGAACATCCCAGTGACG-3 |  |
| *plc*B1 | 5’-CGTCACTGGATGTTCGCATAGCCTACTGGAGCTAGACTA-3 | 861bp |
| *plc*B2 | 5’-GGGTCGACAACCTTGGAATTTATCGCTTG-3 |  |
