# Supplementary figures and images for: A Promising Listeria-Vectored Vaccine Induces Th1-Type Immune Responses and Confers Protection Against Tuberculosis
Source: Front Cell Infect Microbiol. 2017 Sep 28;7:407. doi: 10.3389/fcimb.2017.00407 (PMC5626977; doi:10.3389/fcimb.2017.00407)

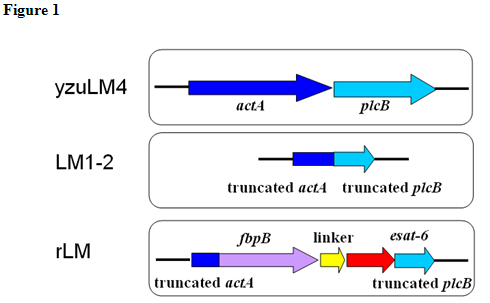

Supplement: Supplementary file 2 [file Image1.jpeg]
